# Imatinib mesylate inhibits cell growth of malignant peripheral nerve sheath tumors *in vitro* and *in vivo* through suppression of PDGFR-β

**DOI:** 10.1186/1471-2407-13-224

**Published:** 2013-05-04

**Authors:** Jun Ohishi, Mikiko Aoki, Kazuki Nabeshima, Junji Suzumiya, Tamotsu Takeuchi, Akira Ogose, Michiyuki Hakozaki, Yuichi Yamashita, Hiroshi Iwasaki

**Affiliations:** 1 Department of Pathology, Fukuoka University School of Medicine, 7-45-1 Nanakuma, Jonan-ku, Fukuoka, 814-0180, Japan; 2 Gastroenterological Surgery, Fukuoka University School of Medicine, Fukuoka, Japan; 3 Shimane University Hospital Cancer Center, Shimane, Japan; 4 Department of Immunopathology, University School of Medicine, Gifu, Japan; 5 Division of Orthopaedic Surgery, Department of Regenerative Transplant Medicine, Niigata University Graduate School of Medicine and Dental Science, Niigata, Japan; 6 Department of Orthopaedic Surgery, Fukushima Medical University School of Medicine, Fukushima, Japan

## Abstract

**Background:**

Malignant peripheral nerve sheath tumors (MPNSTs) are highly aggressive and associated with poor prognosis. Basic research to develop new treatment regimens is critically needed.

**Methods:**

The effects of imatinib mesylate on MPNSTs were examined in six human MPNST cell lines and in a xenograft mouse model.

**Results:**

The results showed expression of platelet-derived growth factor receptor-β and suppression of its phosphorylation by imatinib mesylate in all six cell lines. Imatinib mesylate effectively suppressed MPNST cell growth *in vitro* at concentrations similar to those used clinically (1.46 − 4.6 μM) in three of six cell lines. Knockdown of PDGFR-β by transfection with a specific siRNA also caused significant reduction in cell proliferation in the sensitive cell lines, but not in the resistant cell lines. Furthermore, imatinib mesylate also significantly suppressed colony formation within soft agar and tumor growth in xenograft models using two of the three sensitive MPNST cell lines. There was excellent agreement between *in vitro* and *in vivo* sensitivity to imatinib mesylate, suggesting possible selection of imatinib-sensitive tumors by *in vitro* analysis.

**Conclusions:**

The results suggest that imatinib mesylate may be useful in the treatment of MPNST patients and *in vitro* studies may help select cells that are sensitive to imatinib mesylate *in vivo*.

## Background

Malignant peripheral nerve sheath tumor (MPNST) is an uncommon malignancy defined as malignant tumor arising from a peripheral nerve or showing nerve sheath differentiation, and accounts for about 5% of all soft tissue sarcomas [1,2]. Approximately half of the MPNST are found in patients with neurofibromatosis type 1 (NF1), while the rest develop *de novo* from peripheral nerves. The incidence of MPNST is 0.001% in the general population, but is as high as 2% to 13% in NF1 patients [3,4]. MPNST cells appear to undergo several genetic changes during their progression to the malignant phenotype, although the mechanisms involved in this process remain unknown. MPNST is a very aggressive tumor, with a high rate of local recurrence and distant metastasis. Because chemotherapies and radiation therapies are unsuccessful, surgical removal is presently the only effective treatment [5,6]. Patients with unresectable primary tumors or those with clinically evident metastases have poor prognosis. The reported overall 5- and 10-year survival rates are 34% and 22%, respectively [4]. Thus, there is a need to develop more effective therapeutic modalities for MPNST.

We previously reported the identification of platelet-derived growth factor-BB (PDGF-BB) as an MPNST cell invasion-inducing factor by profiling eight motogenic growth factors in two human MPNST cell lines [7]. We also demonstrated higher mRNA and proteins expression levels of platelet-derived growth factor receptor-β (PDGFR-β) in MPNST tissues than in benign peripheral nerve sheath tumors, such as schwannomas and neurofibromas. The results also showed that PDGF-BB induced tyrosine phosphorylation of PDGFR-β, and that imatinib mesylate inhibited MPNST cell invasion and proliferation by suppressing the phosphorylation of PDGFR-β.

Overexpression of growth factors and/or their receptors is likely to play an important role in cellular transformation, and our previous results suggested that PDGFR-β is a potentially promising target in the design of novel treatments against MPNST. The objective of this study was to analyze the cytotoxic effects of imatinib mesylate on MPNST *in vitro* using six human MPNST cell lines (FU-SFT8611, FU-SFT9817, HS-Sch-2, NMS-2, NMS-2PC, and FMS-1) and its therapeutic effects in a xenograft mouse model.

## Methods

### MPNST cell lines

The six human MPNST cell lines used in this study included FU-SFT8611, FU-SFT9817, HS-Sch-2, NMS-2, NMS-2PC, and FMS-1. The FU-SFT8611 and FU-SFT9817 cell lines were established in our department, as described previously [8]. The HS-Sch-2 cell line was established at Gifu University School of Medicine from a left thigh MPNST in a 54-year-old woman with no clinical evidence of NF1 [9]. The NMS-2 cell line was established at Niigata University from an MPNST in the right thigh of a 30-year-old man with NF1, and NMS-2PC cells were derived from a retroperitoneal metastasis diagnosed 9 months later in the same patient [10]. The patient received pre- and post-operative chemotherapy. The FMS-1 cell line was established at Fukushima Medical University from a right axillary MPNST of a 69-year-old woman with NF1 [11].

FU-SFT8611, FU-SFT9817, and HS-Sch-2 cells were maintained in a 1:1 mixture of Dulbecco’s modified Eagle’s medium (DMEM) (Gibco BRL, Rockville, MD, USA) and Ham’s F12 (Nissui Seiyaku, Tokyo, Japan), while FMS-1, NMS-2, and NMS-2PC cells were cultured in Roswell Park Memorial Institute 1640 (RPMI1640) medium. Both types of media, at pH 7.35, were supplemented with 10% fetal calf serum (FCS), L-glutamine (746 μg/ml), sodium bicarbonate (0.2%), streptomycin (90 μg/ml) and penicillin G (90 μg/ml), used as the growth medium. All cell lines were maintained under a humidified 5% CO_2_ atmosphere at 37°C and the medium was replaced every 3 days.

### Agents

Imatinib mesylate (STI571) was kindly provided by Novartis Pharma AG (Basel, Switzerland). The drug was diluted in sterile distilled water to a stock concentration of 10 mM. The stock solution was stored at −20°C and protected from light. Dilutions of this stock solution were prepared immediately before use in cell culture medium and added directly to the cells. Anti-PDGFR-β, PDGFR-α, phospho-PDGFR-β, phospho-PDGFR-α, and α-tubulin antibodies were purchased from Cell Signaling Technology (Danvers, MA, USA). Recombinant human PDGF-BB was obtained from R&D Systems (Minneapolis, MN, USA).

### Detection of PDGFR and phosphorylated PDGFR by western blotting

All six MPNST cell lines were cultured in growth medium in 25 cm^2^ culture bottles. After stabilization of the cells, the serum-containing medium was aspirated and replaced with serum-free medium for 24 h. After with or without pretreatment for 60 min with 10 μM imatinib mesylate, the cells were stimulated for 30 min, with 25 ng/ml of PDGF-BB in the presence or absence of imatinib mesylate. After removal of the medium, the cells were washed and scraped in phosphate-buffered saline (PBS) and collected by centrifugation at 1,000 rpm for 5 min. The cells were then lysed with a cell lysis buffer consisting of 50 mM Tris–HCl, pH 7.4, 150 mM NaCl, 1 mM ethylenediaminetetraacetic acid (EDTA), 1% Triton X-100, 1 mM Na_3_VO_4_, and protease inhibitor cocktail tablets (Complete Mini, Roche Applied Sciences, Penzberg, Germany). Lysed cells were sonicated on ice for 15 min and centrifuged at 15,000 rpm for 20 min at 4°C. The resultant supernatants were subjected to sodium dodecyl sulfate-polyacrylamide gel electrophoresis (SDS-PAGE) after measurement of their protein concentrations using the Bio-Rad protein assay (Hercules, CA, USA). After electrophoresis, the proteins were transferred electrophoretically to Immobilon membrane (Millipore, Bedford, MA, USA). Non-specific sites were blocked with 2% bovine serum albumin (BSA) in 0.05% Tween-20/Tris-buffered saline, pH 7.6 (TBS-T) at 37°C for 1 h and membranes were incubated overnight at 4°C with antibodies against PDGFR-α, PDGFR-β, phospho-PDGFR-α, phospho-PDGFR-β and α-tubulin dissolved in TBS-T containing 1% BSA. After washing with TBS-T, the membrane was incubated for 1 h with peroxidase-conjugated goat anti-rabbit (for anti-PDGFR-α, phospho-PDGFR-α, and phospho-PDGFR-β and α-tubulin antibodies) or anti-mouse IgG (for anti-PDGFR-β antibody). Color was developed with chemiluminescence reagents according to the instructions supplied by the manufacturer (DuPont NEN, Boston, MA, USA). The bands on the film were subjected to image analysis (Image J version 1.44 software, National Institute of Health, Bethesda, MD, USA). Statistical analysis was performed using the Student’s *t*-test.

### RNA extraction and quantitative real-time reverse transcription-polymerase chain reaction (RT-PCR) analysis

Total RNA was isolated from all six MPNST cell lines using the High Pure RNA Tissue Kit, 2033674 (Roche Applied Science, Indianapolis, IN, USA) according to the instructions supplied by the manufacturer. Next, One microgram of total RNA from each sample was reverse-transcribed using the PrimeScript II 1st standard cDNA Synthesis Kit (Takara Bio, Otsu, Japan). Real-time monitoring of PCR reactions was performed using the Light-Cycler system (Roche Applied Science) and SYBRVRPremix Ex Taq TMII (Takara Bio), according to the instructions supplied by the manufacturer. Gene-specific oligonucleotide primer pairs for PDGFR-β, PDGFR-α and glyceraldehydes-3-phosphate dehydrogenase (GAPDH) were purchased from Takara Bio; PDGFRB forward (F): GCCCTTATGTCGGAGCTGAAGA, reverse (R): GTTGCGGTGCAGGTAGTCCA; PDGFRA (F): CTGACATTGACCCTGTCCCTGA, (R): GATGAAGGTGGAACTGCTGGAAC; and GAPDH (F): GCACCGTCAAGGCT GAGAAC, (R): TGGTGAAGACGCCAGTGGA.

### Cytotoxicity assay

MPNST cell proliferation was evaluated using the One-Solution Cell Proliferation Assay with the tetrazolium compound 3-(4,5-dimethylthiazol-2-yl)-5-(3- carboxymethoxyphenyl)-2-(4-sulfophenyl)-2H-tetrazolium, inner salt (MTS) (CellTiter 96 Aqueous, Promega, Madison, WI, USA). The MTS compound is bioreduced to formazan by reduced NADPH or reduced NADH produced by metabolically active dehydrogenases of cells, which can be detected at 490 nm. After treatment of MPNST cells with 100 μl medium in each well of a flat-bottomed 96-well plate, 20 μl of MTS solution was added to each well and incubated at 37°C for 1 h. The 96-well plate was then placed in a kinetic microplate reader (Benchmark, Bio-Rad) and absorbance was measured at 490 nm.

The effects of imatinib mesylate on MPNST cell proliferation and survival were determined in 96-well plates by the MTS assay. The six MPNST cell lines were seeded onto the wells of flat-bottomed 96-well plate at each appropriate number for proliferation (FU-SFT8611 and FU-SFT9817 cells: 1 × 10^3^ cells per well, HS-Sch-2, NMS-2, NMS-2PC and FMS-1 cells: 3 × 10^3^ cells per well) in growth medium. Cells were allowed to adhere to the substratum overnight and then the medium was replaced by treatment medium containing 2% FCS in the presence of different concentrations of imatinib mesylate (1, 5, 10 and 20 μM) (day 0). The control groups were incubated with fresh imatinib mesylate-free 2% FCS containing medium. The test medium containing 2% FCS without or with different concentrations of imatinib mesylate was changed every 2 days. At days 0, 1, 3, and 5 of the experiment, the cell proliferation was determined by the MTS assay.

### Small interfering RNA (siRNA)

MPNST cells were seeded in 6-well plates in antibiotic-free medium containing 10% FCS at a density of 1.5 × 10^5^ cells per well. After stabilization of the cells overnight, the cells were transfected with pooled siRNA for PDGFR-βor negative control siRNA (B-Bridge International, Sunnyvale, CA, USA) using LipofectAMIN 2000 (Invitrogen, Carlsbad, CA) according to the instructions supplied by the manufacturer. After 72 h transfection, the cells were lysed and analyzed by western blotting with anti-PDGFR-β, PDGFR–α, and α-tubulin antibodies to confirm knock down at the protein level. To examine the effect of siRNA for PDGFR-β on proliferation of MPNST cells, cells were similarly transfected with siRNA in flat-bottomed 96-well plate (3 × 10^3^ cells per well). Growth medium containing 2% FCS was replaced every 72 h, followed by assessment of cell proliferation at days 1, 3, 5, and 7 using MTS assays as described above.

### Soft agar colony formation assay

The effect of imatinib mesylate on the anchorage-independent growth of 3 MPNST cell line was examined using a soft agar colony formation assay. After the addition of 50 μl per well of 0.6% agar solution containing 10% FCS containing medium to the bottom of flat-bottomed 96-well plate, as a basal agar layer, 75 μl per well of 0.4% agar solution with 7% FCS containing 6.5 × 10^3^ MPNST cells were transferred onto the basal agar layer as a cell agar layer. Imatinib mesylate was added to the cell agar layer at final concentrations of 5 or 10 μM. After solidification of the cell agar layer, 2% FCS medium (100 μl) with or without imatinib mesylate (5 or 10 μM) was overlaid onto the layers. After 7-days incubation at 37°C, the numbers of anchorage-independent tumor cell colonies growing in the soft agar were counted using a phase contrast microscope.

### Animal xenograft model and treatment with imatinib mesylate

The experimental protocol was approved by the Ethics Review Committee for Animal Experimentation of Fukuoka University School of Medicine. In these experiments, 5 × 10^6^ cells from each of three MPNST cell lines, HS-Sch-2, FMS-1 and NMS-2PC, were transplanted into the subcutis of the right flank of 6–8 week-old female NOD/SCID mice. When the tumor size reached 100 mm^3^ (about 28 days after cell inoculation), the mice were randomly divided into two groups: one group received water (control group), and the other was treated orally with imatinib mesylate (100 mg/kg/day) (treatment group). The latter group was closely watched for unusual symptoms or behaviors in order to evaluate systemic toxicity of imatinib mesylate, and the body weight was measured once a week.

Tumor size was measured once a week. The tumor volume was calculated according to the formula: L × W^2^ × 0.5, where L is the longest diameter and W is the width. At each time point, the mean tumor volume was compared between the two groups with a two-tailed Student’s *t*-test. The level of statistical significance was set at *P* < 0.05. After euthanasia, the tumors and internal organs were harvested and immediately fixed in 10% formalin. Non-necrotic portions of the tumor were excised and frozen. Various organs were prepared for histopathological analysis.

### DNA extraction, PCR and DNA sequencing

Genomic DNA was extracted from the cell lines by using a GenElute Mammalian Genomic DNA Miniprep Kit (Sigma-Aldrich, St. Louis, MO, USA) based on the procedure recommended by the manufacturer, and *PDGFR-β* exons 12 and 18 were analyzed by PCR as described previously [7]. The primer sequences used were as follows; *PDGFR-β* exon 12 primer forward (F): TGTCCTAGACGGACGAACCT, reverse (R): CCAACTTGAGTCCCCACACT; exon 18 (F): GAAGGGTCTTTCCCCACAAT, (R): CACACTGGTCAGGAGGGAAT. The PCR products were purified using a QiAquick PCR purification kit (Qiagen Inc., Hilden, Germany). Direct sequencing of PCR products was performed using Applied Biosystems 3730 DNA Analyzer (Applied Biosystems, Foster City, CA, USA).

### Fluorescence in situ hybridization (FISH)

FISH of 6 MPNST cell lines was performed by labeling bacterial chromosomes (BACs) centromeric (RP11-1149B8 and RP11-348I17) and telomeric (RP11-101B10 and RP11-434E5) to the PDGFB with Spectrum Green and Spectrum Red (Abbott Molecular Inc., Des Plaines, IL, USA). Detection of the labeled probes with Spectrum Red and Spectrum Green was performed with streptavidin Alexa 594 (Molecular Probes, Eugene, OR, USA) and fluorescein isothiocyanate anti-diogoxigenin (Roche).

## Results

### Expression of PDGFR-β and PDGFR–α in six MPSNT cell lines

**Figure 1:**
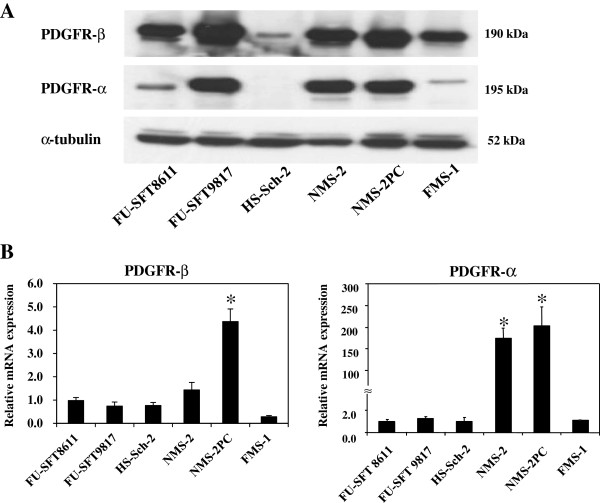
**Expression of PDGFR proteins and mRNAs of in six MPNST cell lines analyzed by western blotting (A) and real-time RT-PCR (B).** The mRNA expression levels in each cell line were compared with the levels in FU-SFT8611 cells. PDGFR-β protein and mRNA were expressed in all MPNST cell lines. The expression of PDGFR-α protein was observed in all but HS-Sch-2 cells. The PDGFR-α mRNA level in NMS-2 and NMS-2PC cells was higher than in the other four cell lines. Data in (**B**) is the mean ± SEM (n = 3). Similar results were obtained in three independent experiments. *P < 0.01, compared with the level in FU-SFT8611 cells (by Student’s *t*-test).

Western blotting detected the expression of PDGFR-β protein in all six of the MPNST cell lines, and the expression of PDGFR-α protein in all but the HS-Sch-2 cells (Figure 1A). Quantitative real-time RT-PCR identified the expression of both PDGFR-α and PDGFR–β mRNAs in all six of the MPNST cell lines. Interestingly, the NMS-2 and NMS-2PC cell lines expressed both mRNAs at higher levels than the other cell lines (Figure 1B).

### Effects of PDGF-BB and imatinib mesylate on PDGFR-β phosphorylation *in vitro*

**Figure 2:**
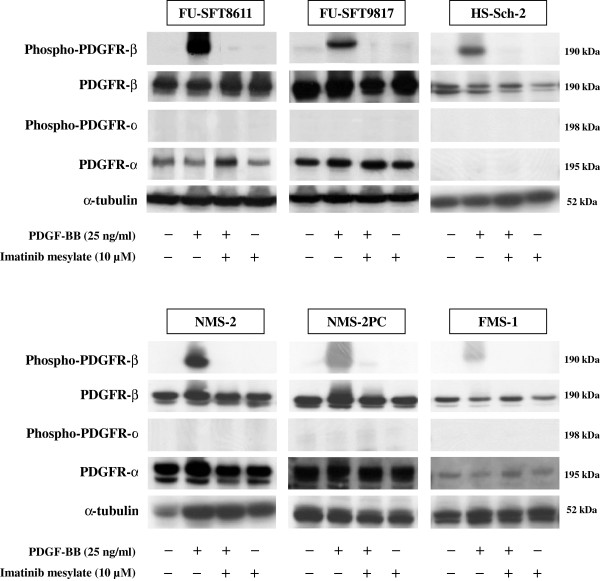
**Effects of PDGF-BB and imatinib mesylate on phosphorylation of PDGFRs.** PDGFR-β of all six MPNST cell lines was phosphorylated in response to 25 ng/ml of PDGF-BB for 30 min. PDGF-BB stimulation did not induce any phosphorylation of PDGFR-α in all cell lines. Pretreatment with 10 μM imatinib mesylate almost completely suppressed PDGF-BB-induced PDGFR-β phosphorylation in all cell lines.

The effects of PDGF-BB and imatinib mesylate on the phosphorylation of PDGFRs were investigated. In all six of the MPNST cell lines, PDGF-BB induced phosphorylation of PDGFR-β, but not of PDGFR-α (Figure 2). Furthermore, pretreatment with imatinib mesylate (10 μM) almost completely suppressed PDGF-BB-induced phosphorylation of PDGFR-β in all six cell lines.

### Effects of imatinib mesylate on MPNST cell proliferation

**Figure 3:**
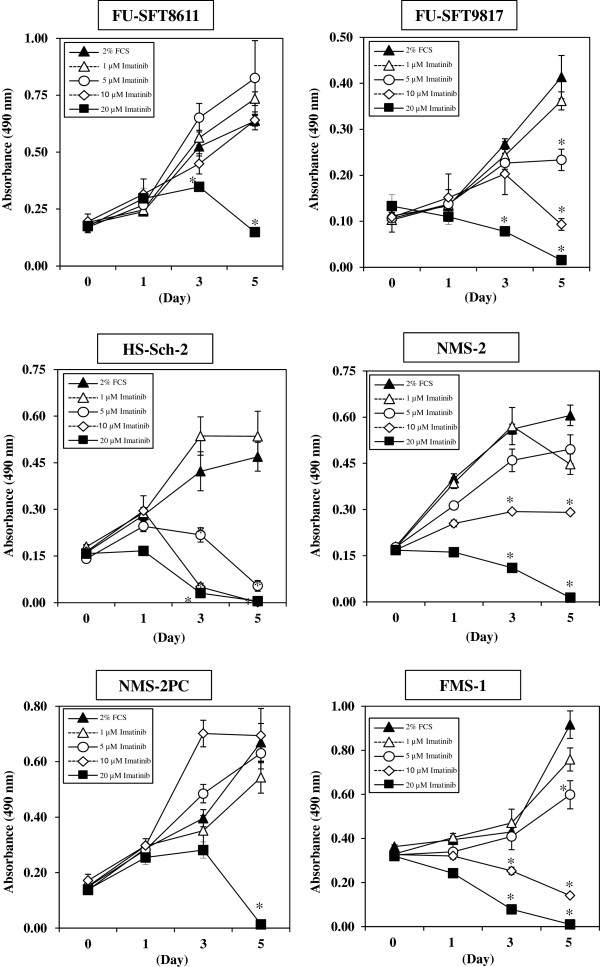
**Effects of imatinib mesylate on the proliferation of six MPNST cell lines.** MPNST cells were incubated for 5 days with imatinib mesylate at the indicated concentrations in media containing 2% FCS. One to five μM of imatinib mesylate had significant inhibitory effects at day 5 in three cell lines, FU-SFT9817, HS-Sch-2, and FMS-1 cells. Data are the means ± SEM (n = 4). *P < 0.01, compared with the control group without imatinib mesylate (by Student’s *t*-test).

The effects of imatinib mesylate on MPNST cell proliferation were tested in all six MPNST cell lines at concentrations of 1, 5, 10, and 20 μM for 5 days, using the MTS assay (Figure 3). One to five μM of imatinib mesylate, which are equivalent to the concentrations achieved clinically in patients treated with imatinib mesylate, had a significant inhibitory effect in three cell lines (FU-SFT9817, HS-Sch-2, and FMS-1 cells) at day 5, only a mild inhibitory effect in NMS-2 cells, but no effect in FU-SFT8611 or NMS-2PC cells. The 50% inhibitory concentration (IC_50_) of imatinib mesylate at day 5 for FU-SFT8611, FU-SFT9817, HS-Sch-2, NMS-2, NMS-2PC, and FMS-1 cells were 16.6, 4.2, 3.2, 4.7, 14.7, and 4.2 μM, respectively.

### Effects of siRNA-mediated inhibition of PDGFR-β expression on MPNST cell growth

**Figure 4:**
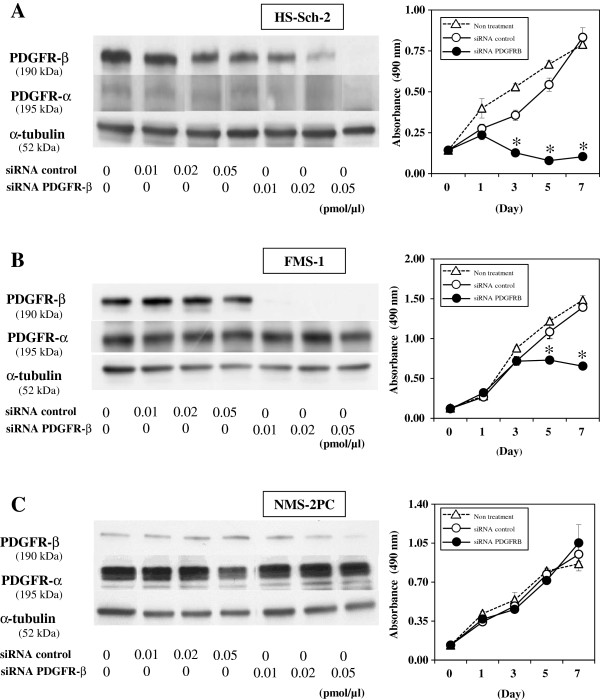
**Effects of siRNA-mediated inhibition of PDGFR-β expression on the growth of 3 MPNST cell lines.** After 72 h transfection with 0.01, 0.02 and 0.05 pmol/μl siRNA, specific PDGFR-β knockdown was induced in all three cell lines. In the presence of 0.02 pmol/μl siRNA for PDGFR-β, cell proliferation was significantly inhibited compared with the control, in two MPNST cell lines, HS-Sch-2 (**A**) and FMS-1 (**B**), but not NMS-2PC (**C**). Data are the means ± SEM (n = 5). Similar results were obtained in at least two independent experiments. *P < 0.01 (by Student’s *t*-test).

To clarify the role of PDGFR-β in MPNST cell proliferation, we examined the effects of siRNA for PDGFR-β on cell proliferation in the two imatinib mesylate-sensitive cell lines (HS-Sch-2 and FMS-1), and on one resistant cell line (NMS-2PC). Transfection with PDGFR-β siRNA specifically and effectively abrogated PDGFR-β protein expression in all three cell lines (Figure 4). The specific PDGFR-β knockdown significantly inhibited HS-Sch-2 and FMS-1 cell proliferation (Figure 4A, B), whereas NMS-2PC cells showed no suppression of cell proliferation (Figure 4C).

### Effects of imatinib mesylate on colony formation

**Figure 5:**
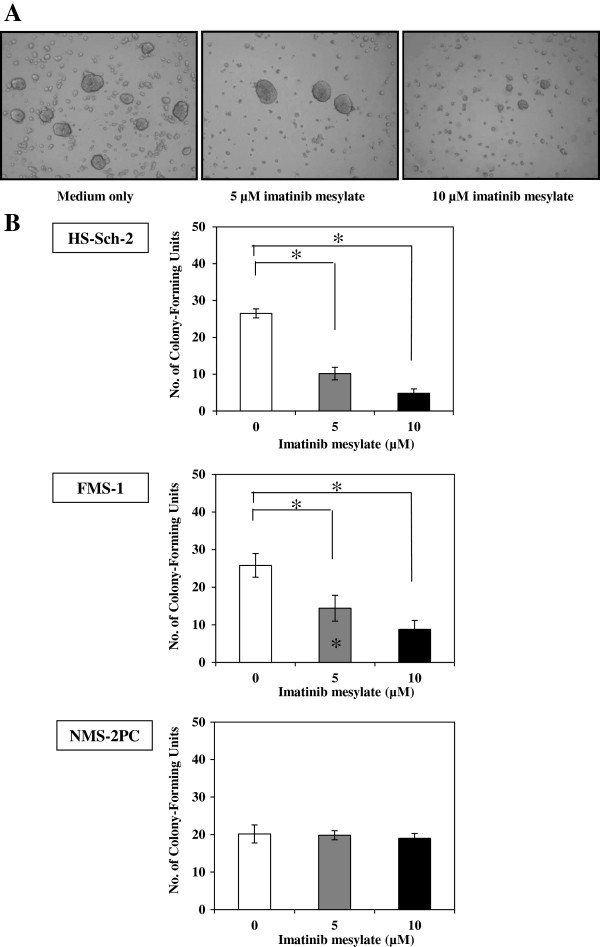
**Soft agar colony formation assay.** Anchorage-independent growth in soft agar was examined under different concentrations of imatinib mesylate, and numbers of colonies were counted at day 7. (**A**) Numbers of anchorage-independent colonies of HS-Sch-2 cells growing in the soft agar were scored using phase contrast microscopy. (**B**) Imatinib mesylate at 5 μM significantly decreased the number of colonies of HS-Sch-2 and FMS-1 cells, but had no effect on NMS-2PC cells. Data are the means ± SEM (n = 6). *P < 0.01, compared with the control group without imatinib mesylate (by Student’s *t*-test).

In the anchorage-independent growth examined using the soft agar colony formation assays, both imatinib mesylate-sensitive cell lines (HS-Sch-2 and FMS-1) were also responsive to imatinib mesylate (Figure 5A, B). Treatment with both 5 and 10 μM imatinib mesylate significantly inhibited anchorage-independent growth in these two cell lines, whereas the NMS-2PC cell line was resistant to both concentrations of imatinib mesylate.

### Effects of imatinib mesylate on MPNST growth in the xenograft model

**Figure 6:**
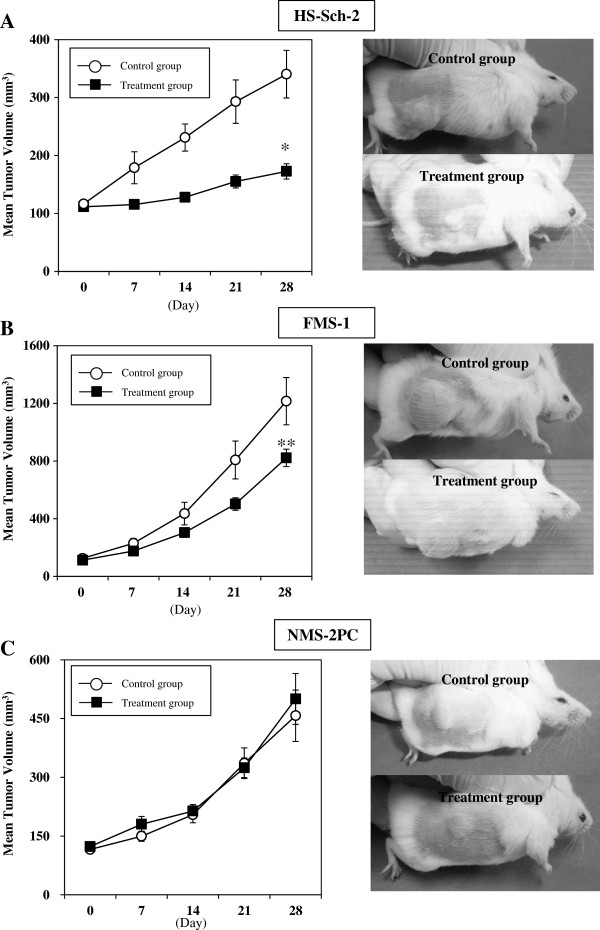
**Effects of oral treatment with imatinib mesylate on tumor growth in mice of the xenograft model.** One group of mice was treated with 100 mg/kg/day imatinib mesylate (■; treatment group) while the other was treated with water (○; control group). (**A**, left) Xenografts were established by injection of HS-Sch-2 cells in female NOD/SCID mice. The tumor volume was significantly smaller in the treatment group than the control (■; n = 5, ○; n = 6, *P < 0.01, by Student’s *t*-test) on day 28. (**B**, left) Imatinib mesylate significantly reduced the growth of FMS-1 xenografts compared with the control groups (■; n = 7, ○; n = 6, **P < 0.05, by Student’s *t*-test). (**C**, left) Imatinib mesylate did not affect the growth of NMS-2PC xenografts (■; n = 4, ○; n = 4). Representative picture of animals are shown at right for each experiment.

To investigate the inhibitory effect of imatinib mesylate on MPNST growth *in vivo*, three MPNST cell lines, two imatinib mesylate-sensitive, HS-Sch-2 and FMS-1 cells, and one -resistant, NMS-2PC cells, were each transplanted subcutaneously into separate NOD/SCID mice (n = 5-7, each). In HS-Sch-2 and FMS-1 cell-transplanted mice, daily treatment with imatinib mesylate (100 mg/kg/day) significantly suppressed tumor growth compared with the control group (*P* < 0.01 for HS-Sch-2 group; *P* < 0.05 for FMS-1 group. Figure 6A, B). In contrast, imatinib mesylate did not suppress tumor growth in mice bearing NMS-2PC cells (Figure 6C).

During imatinib mesylate treatment, the body weight was only slightly affected, with less than 7% weight reduction in all mice (Additional 1: Figure S1). Skin dehydration, diarrhea, or abnormal behaviors were not observed. Histopathologically, there were no apparent differences between the treatment group and the control group, such as inflammatory infiltrates, necrotic foci, the number of intratumoral blood vessels, or degenerated cell changes (Additional 2: Figure S2). There were no differences in histological findings of the intestine, spleen, liver, and lungs between the treatment and control groups (Additional 3: Figure S3).

### Mutation of the PDGFRB gene and chromosomal rearrangement including the PDGF-B gene

Because it is well known that the mutations of tyrosine kinase receptors affect the effectiveness of tyrosine kinase inhibitors, we analyzed mutation in exons 12 and 18 of the *PDGFRB* gene in all six MPNST cell lines. These exons correspond to the mutation-positive juxtamembrane and activation loop coding regions of the *KIT* and *PDGFRA* genes [12]. Using FISH, we also examined whether MPNST cell lines contained fusion genes involving the *PDGF-B* gene as observed in dermatofibrosarcoma protuberans (DFSP) [13]. Neither mutation in exons 12 and 18 of the *PDGFRB* nor split of the *PDGF-B* gene was detected in any of the 6 MPNST cell lines (Additional 4: Figure S4).

## Discussion

The present study is the first to show that imatinib mesylate effectively suppressed cell growth *in vitro* at concentrations within the therapeutic range (1.46-4.6 μM), in three of the six human MPNST cell lines. In two of these three imatinib mesylate-sensitive cell lines (HS-Sch-2 and FMS-1), we confirmed that imatinib mesylate also significantly suppressed tumor growth in the xenograft model. There was excellent concordance between *in vitro* and *in vivo* sensitivity to imatinib mesylate.

In the imatinib mesylate-sensitive cell lines, imatinib mesylate suppressed MPNST cell growth via inhibition of PDGFR-β phosphorylation. Knockdown of PDGFR-β by transfection with a specific siRNA also caused significant reduction in cell proliferation in the sensitive cell lines but not in resistant cell lines. Furthermore, although PDGF-BB-induced phosphorylation of PDGFR-β was inhibited by imatinib mesylate in all cell lines, cell growth was suppressed by imatinib mesylate only in the imatinib mesylate-sensitive cell lines. These results support the conclusion that PDGFR-β is involved in the main intracellular signal pathway for tumor cell growth in imatinib mesylate-sensitive cell lines.

PDGF was first identified as a growth-promoting factor produced by human platelets for mesenchymal cells such as dermal fibroblasts, smooth muscle cells, and other connective tissue cells [14]. PDGF consists of four chains (A, B, C, and D), which dimerize to form at least five hetero- and homo-dimers: PDGF-AA, PDGF-AB, PDGF-BB, PDGF-CC, and PDGF-DD [15]. PDGFR is a transmembrane tyrosine kinase receptor and there are two receptor types, termed PDGFR-α and PDGFR-β [16]. The two different subunits of PDGFR have different PDGF dimers binding specificities. Based on cell culture experiments, the possible PDGF/PDGFR interactions are multiple and complex: PDGFR-αα binds PDGF-AA, PDGF-AB, PDGF-BB and PDGF-CC; PDGFR-ββ binds PDGF-BB, and PDGF-DD; and PDGFR-αβ binds PDGF-AB, PDGF-BB, PDGF-CC, and PDGF-DD. However, *in vivo*, only a few interactions were demonstrated: PDGFR-αα binds PDGF-AA and PDGF-CC, and PDGFR-ββ binds only PDGF-BB [15]. In the present study, like the previous study [7], PDGF-BB induced tyrosine phosphorylation of PDGFR-β but not of PDGFR-α, although both types of receptors were expressed in all but the HS-Sch-2 cell line. These results suggest that activation of PDGFR-β may play a more important role in MPNST cell proliferation than PDGFR-α. The observation that PDGFR-β mRNA and protein expression were higher in MPNST than in benign peripheral nerve sheath tumors or Schwann cells [7,17], also supports its important role.

It is well known that mutations of tyrosine kinase receptors affect the effectiveness of tyrosine kinase inhibitors, including imatinib mesylate [18]. Most patients with advanced gastrointestinal stromal tumors carry mutations in *c-kit* or *PDGFR-α,* and the response to imatinib mesylate in these patients depends on the type of mutation. To our knowledge, *PDGFRB* mutations have never been described in MPNST, although some somatic *PDGFRA* mutations and several polymorphisms have been reported [19]. We analyzed the sequences of exons 12 and 18 of the *PDGFRB* gene in all six MPNST cell lines, but no *PDGFRB* mutations were detected. We also examined the presence of chromosomal rearrangements including in the *PDGFB* gene, because the fusion gene leads to autocrine PDGF receptor stimulation in dermatofibrosarcoma protuberans (DFSP) [13]. However, no split of the *PDGFB* gene was detected by FISH in all six MPNST cell lines (Additional 4: Figure S4).

The PDGF/PDGFR autocrine loop has an important role in other malignancies, such as glioma [20], osteosarcoma [21], breast cancer [22], and ovarian cancer [23], but has yet to be completely described for MPNST cells. The loop may be involved in the sensitivity of MPNST cells to Imatinib, and we are currently exploring this possibility.

## Conclusions

These results suggest that imatinib mesylate may be useful in the treatment of MPNST patients. When primary cultures of MPNST are available, analysis of sensitivity to imatinib *in vitro* may help select patients with imatinib-sensitive tumors.

## Competing interests

All authors have no conflicts of interest to declare.

## Authors’ contributions

JO, MA carried out the experiments. KN, HI, JS, and YY participated in the design of the study, its coordination, and helped to draft the manuscript. In addition technical guidance from TT, AO, and MH lead to successful experiments. All authors approved the final manuscript.

## Pre-publication history

The pre-publication history for this paper can be accessed here:

http://www.biomedcentral.com/1471-2407/13/224/prepub

## Supplementary Material

Additional file 1: Figure S1 During imatinib mesylate treatment, the body weight was only slightly affected, with less than 7% reduction in all mice. Click here for file

Additional file 2: Figure S2 Transplanted tumors showed a proliferation of atypical spindle or polygonal shaped cells with oval nuclei and distinct nucleoli. These cells proliferated loosely as interconnected cords or networks, or compactly in a sheet-like pattern. Mitotic figures are frequently observed. There were no differences in histological findings between the treatment and control groups. Click here for file

Additional file 3: Figure S3 During imatinib mesylate treatment, there were no differences in histological findings of the intestine, spleen, liver, and lungs between the treatment group and the control group. Click here for file

Additional file 4: Figure S4 Using fluorescence in situ hybridization (FISH), we examined whether MPNST cell lines contained fusion genes involving the *PDGF-B*. No slit of the *PDGF-B* gene was detected in any of the six MPNST cell lines. Click here for file
