# Supplementary material for: Imatinib mesylate inhibits cell growth of malignant peripheral nerve sheath tumors in vitro and in vivo through suppression of PDGFR-β
Source: BMC Cancer. 2013 May 4;13:224. doi: 10.1186/1471-2407-13-224 (PMC3654969; doi:10.1186/1471-2407-13-224)
Supplement: Additional file 1: Figure S1 — During imatinib mesylate treatment, the body weight was only slightly affected, with less than 7% reduction in all mice. [file 1471-2407-13-224-S1.pptx]

## Slide 1
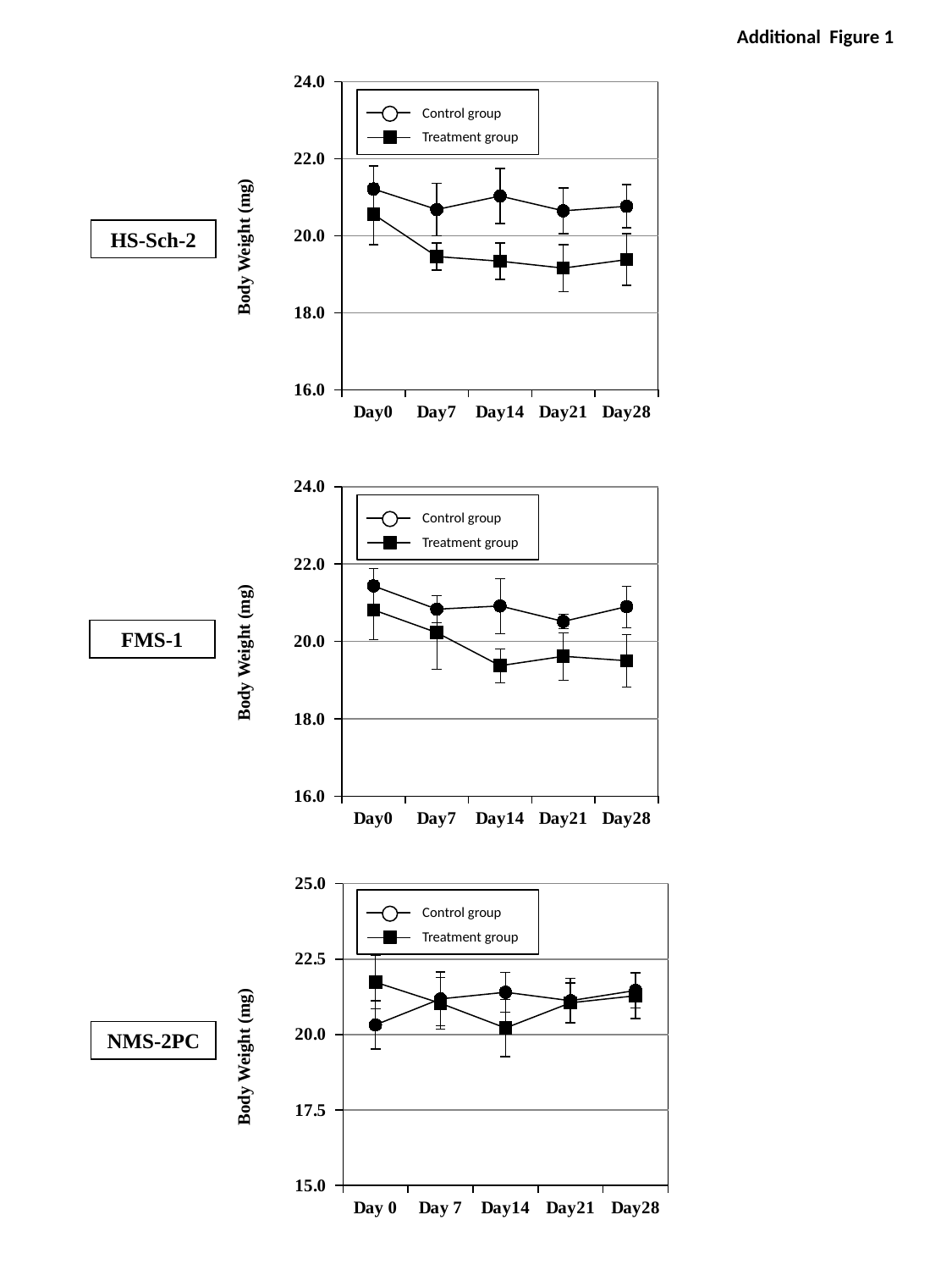

Additional Figure 1
### Chart
| Category | コントロール群 | グリベック 100mg/kg/day |
|---|---|---|
| Day0 | 21.216666666666665 | 20.56 |
| Day7 | 20.683333333333234 | 19.459999999999987 |
| Day14 | 21.033333333333243 | 19.339999999999996 |
| Day21 | 20.65000000000003 | 19.159999999999997 |
| Day28 | 20.766666666666666 | 19.380000000000003 |
Control group
Treatment group
HS-Sch-2
Body Weight (mg)
### Chart
| Category | コントロール群 | グリベック100mg/kg/day群 |
|---|---|---|
| Day0 | 21.433333333333234 | 20.81428571428579 |
| Day7 | 20.833333333333254 | 20.228571428571428 |
| Day14 | 20.916666666666668 | 19.37142857142857 |
| Day21 | 20.51666666666669 | 19.614285714285796 |
| Day28 | 20.900000000000002 | 19.5 |
Control group
Treatment group
FMS-1
Body Weight (mg)
### Chart
| Category | コントロール群 | グリベック(100mg/kg/day)群 |
|---|---|---|
| Day 0 | 20.32 | 21.733333333333242 |
| Day 7 | 21.18 | 21.03333333333325 |
| Day14 | 21.4 | 20.216666666666665 |
| Day21 | 21.119999999999997 | 21.05 |
| Day28 | 21.459999999999987 | 21.28333333333321 |
Control group
Treatment group
NMS-2PC
Body Weight (mg)
