# Supplementary figures and images for: Imatinib mesylate inhibits cell growth of malignant peripheral nerve sheath tumors in vitro and in vivo through suppression of PDGFR-β
Source: BMC Cancer. 2013 May 4;13:224. doi: 10.1186/1471-2407-13-224 (PMC3654969; doi:10.1186/1471-2407-13-224)

## Slide 1
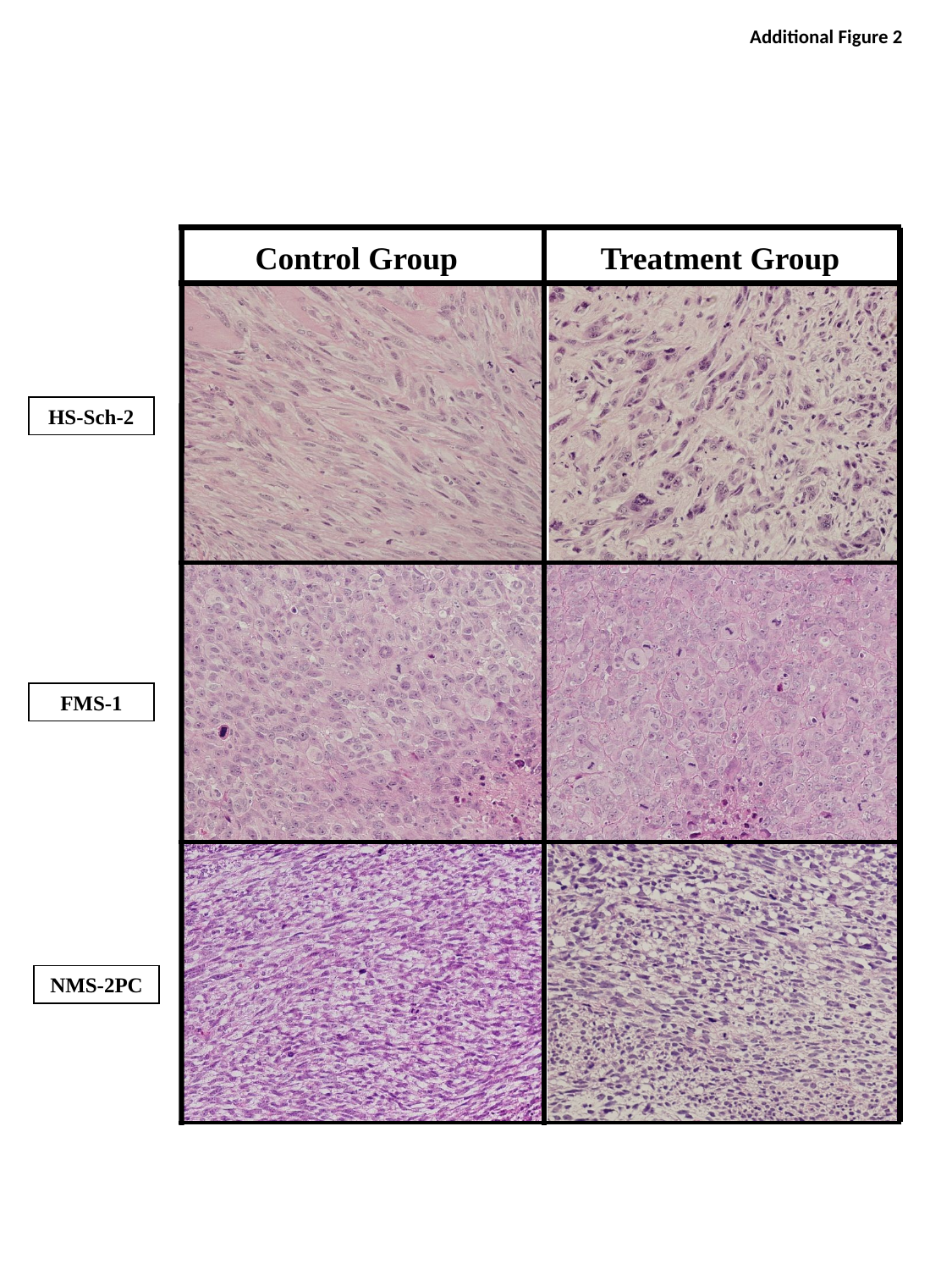

Additional Figure 2
Control Group
Treatment Group
HS-Sch-2
FMS-1
NMS-2PC

Supplement: Additional file 2: Figure S2 — Transplanted tumors showed a proliferation of atypical spindle or polygonal shaped cells with oval nuclei and distinct nucleoli. These cells proliferated loosely as interconnected cords or networks, or compactly in a sheet-like pattern. Mitotic figures are frequently observed. There were no differences in histological findings between the treatment and control groups. [file 1471-2407-13-224-S2.pptx]

## Slide 1
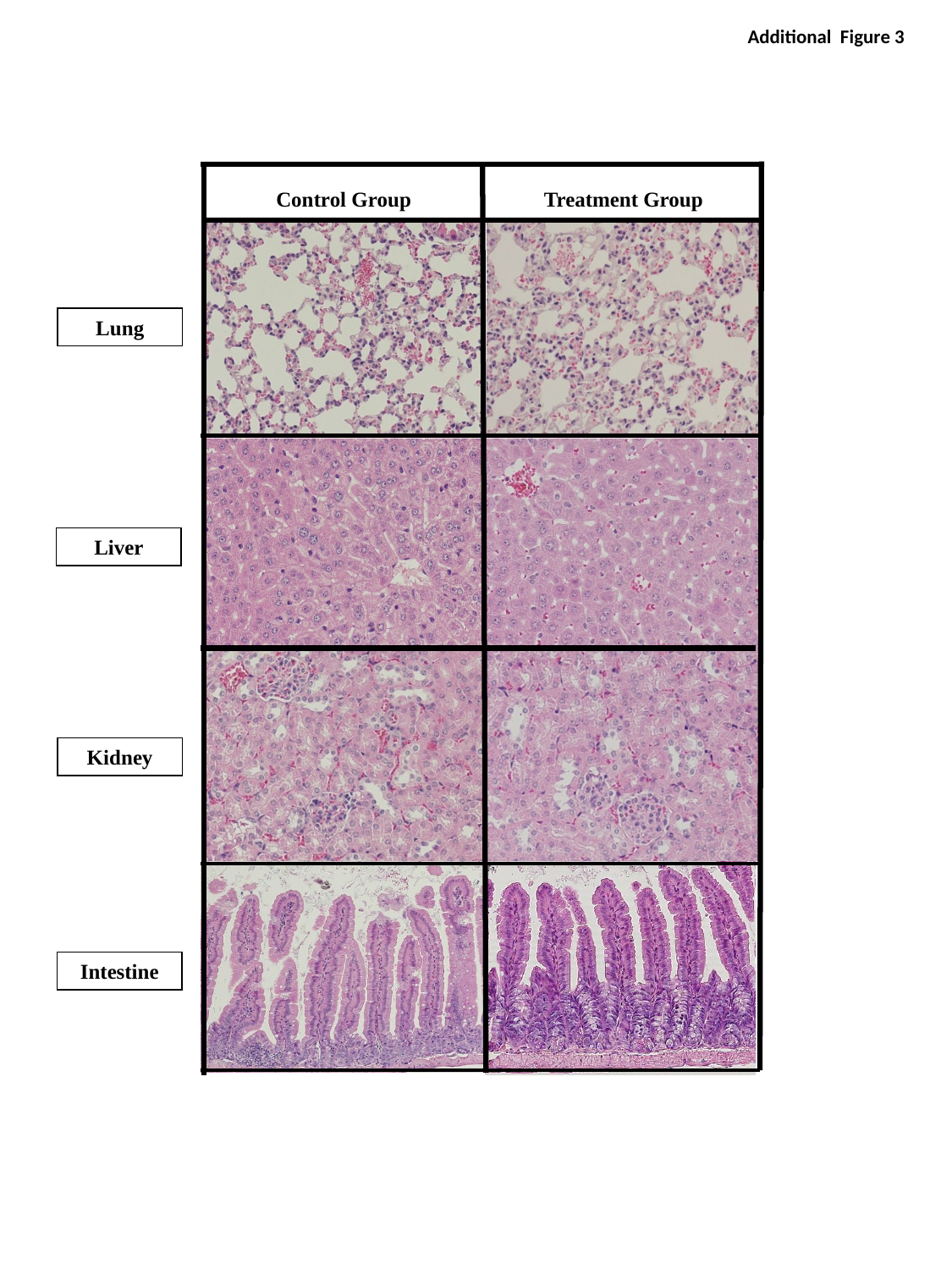

Additional Figure 3
Control Group
Treatment Group
Lung
Liver
Kidney
Intestine

Supplement: Additional file 3: Figure S3 — During imatinib mesylate treatment, there were no differences in histological findings of the intestine, spleen, liver, and lungs between the treatment group and the control group. [file 1471-2407-13-224-S3.pptx]

## Slide 1
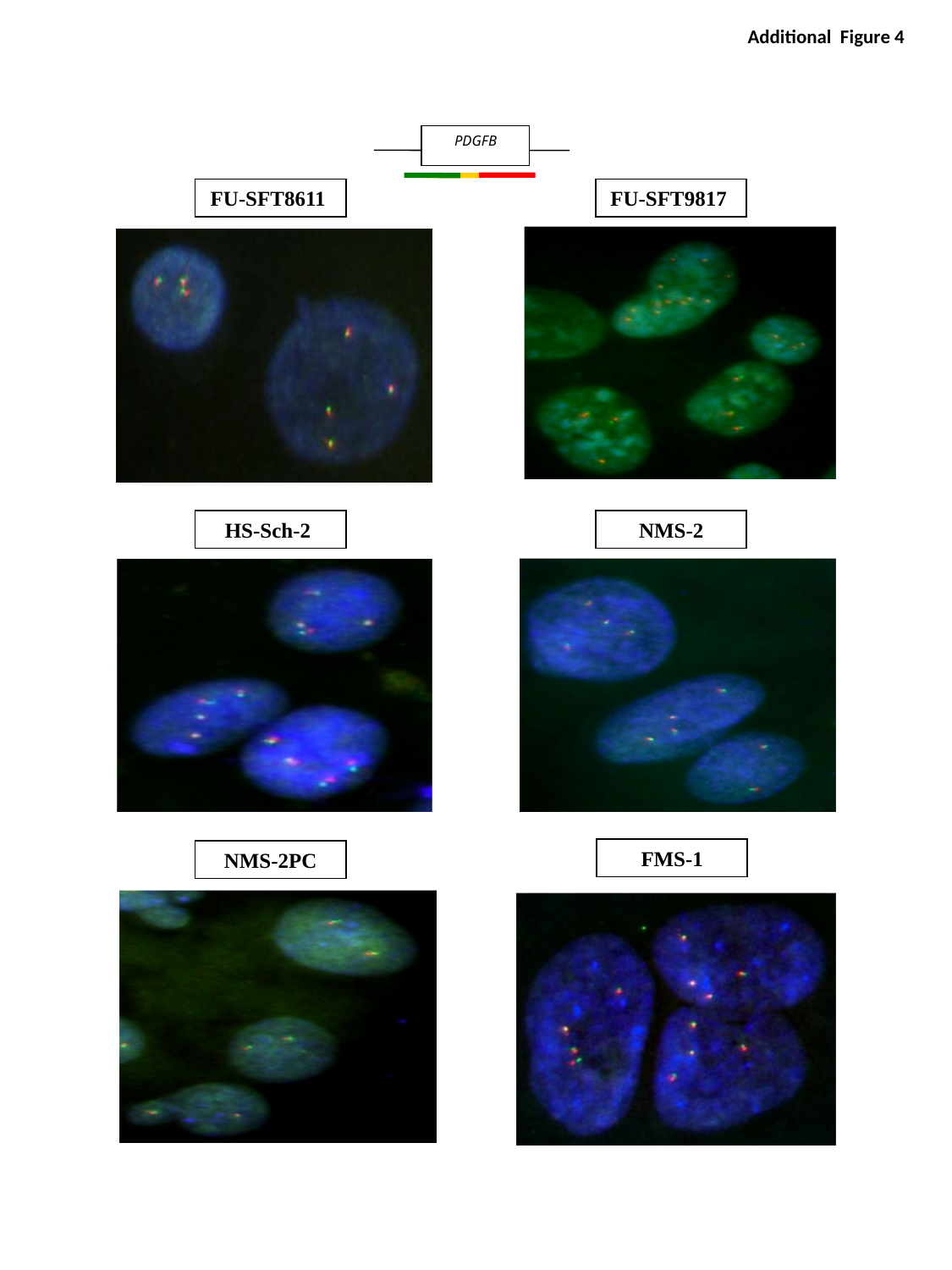

Additional Figure 4
PDGFB
FU-SFT8611
HS-Sch-2
NMS-2PC
FU-SFT9817
NMS-2
FMS-1

Supplement: Additional file 4: Figure S4 — Using fluorescence in situ hybridization (FISH), we examined whether MPNST cell lines contained fusion genes involving the PDGF-B. No slit of the PDGF-B gene was detected in any of the six MPNST cell lines. [file 1471-2407-13-224-S4.pptx]
